# Recirculating packed-bed biofilm photobioreactor combined with membrane ultrafiltration as advanced wastewater treatment

**DOI:** 10.1007/s11356-023-27309-2

**Published:** 2023-05-04

**Authors:** Oliver Díaz, Enrique González, Luisa Vera, Luis Javier Fernández, Ana R. Díaz-Marrero, José J. Fernández

**Affiliations:** 1 Departamento de Ingeniería Química y Tecnología Farmacéutica, Facultad de Ciencias, Universidad de La Laguna, Avenida Astrofísico Francisco Sánchez s/n, 38206 La Laguna, Spain; 2 Instituto de Productos Naturales y Agrobiología (IPNA)-CSIC, Avenida Astrofísico Francisco Sánchez 3, 38206 La Laguna, Spain; 3 Instituto Universitario de Bio-Orgánica Antonio González (IUBO AG), Universidad de La Laguna, Avenida Astrofísico Francisco Sánchez 2, 38206 La Laguna, Spain; 4 Departamento de Química Orgánica, Universidad de La Laguna, Avenida Astrofísico Francisco Sánchez 2, 38206 La Laguna, Spain

**Keywords:** Packed-bed photobioreactor, Microalgal-bacterial consortium, Algal biofilm, Membrane fouling, Nutrient removal, Water reuse

## Abstract

Packed-bed biofilm photobioreactor combined with ultrafiltration membrane was investigated for intensifying the process for secondary wastewater effluent treatment. Cylindrical glass carriers were used as supporting material for the microalgal-bacterial biofilm, which developed from indigenous microbial consortium. Glass carriers allowed adequate growth of the biofilm with limited suspended biomass. Stable operation was achieved after a start-up period of 1000 h, where supernatant biopolymer clusters were minimized and complete nitrification was observed. After that time, biomass productivity was 54 ± 18 mg·L^−1^·day^−1^. Green microalgae *Tetradesmus obliquus* and several strains of heterotrophic nitrification–aerobic denitrification bacteria and fungi were identified. Combined process exhibited COD, nitrogen and phosphorus removal rates of 56 ± 5%, 12 ± 2% and 20 ± 6%, respectively. Membrane fouling was mainly caused by biofilm formation, which was not effectively mitigated by air-scouring aided backwashing.

## Introduction

Water reuse is incorporated in the ‘Circular Economy’ which pursues to close the life cycle of products, services, waste, materials, water and energy and its relevant role on Sustainable Development Goals and Climate Change Adaptation policies and actions is absolutely incontestable.

Nowadays, the most established and implemented technologies for wastewater reclamation in wastewater treatment plants (WWTPs) are the activated sludge process and, more recently, the membrane bioreactors (MBRs). Both of them consume large amounts of energy and materials to comply with discharge and reclamation standards. Nevertheless, MBR represents an advantageous technical in order to reuse wastewater, being very compact and efficient for separation of suspended and colloidal matter and enabling to achieve higher quality effluents, virtually disinfected. The main MBR handicap is high energy consumption, which is usually in the range 0.6–2.3 kWh/m^3^ of treated effluent, depending on the size of the plant and its degree of optimization (Zsirai et al 2012).

In order to operate in a more sustainable mode the wastewater treatment plants (WWTPs), a new paradigm is reconverting these ones to recovery resource facilities. In the case of nutrients, complete or partial removal has been traditionally promoted to prevent eutrophication or aquifer pollution by high levels of nitrogen and phosphorus compounds in the discharge of reclaimed wastewater. By contrary, nowadays, several expertise voices put the attention on developing strategies and technologies able of remaining adequate levels, non-toxic and in accordance to guidelines of nitrogen or phosphorus in water to be reused for irrigation since it is an added value of water reuse. But also, these strategies must comprise alternative options to recover the excess of nutrients not directly assumed by crops. It is not denied the potential of wastewater as feedstock stock. In this sense, Muñoz and Guieysse (2006) support the application of microalgae for their known ability to remove nutrients, such as inorganic nitrogen and phosphorus, heavy metals, and some toxic and organic pollutants in wastewater treatment. Also, the chemical and biochemical oxygen demand can be decreased by microalgae, inhibiting the growth of coliform bacteria (de-Bashan and Bashan 2010) or even showing a mild disinfecting effect, owing to their increased pH during photosynthesis (Petrovič and Simonič 2015). In this sense, integration of the microalgae biomass cultivation with wastewater treatment is a promising approach for developing sustainable treatment technologies.

On the other hand, microalgal-based wastewater treatment systems are less expensive and more efficient for nutrient removal than conventional tertiary treatment, with the added benefits of resource recovery (Christenson and Sims 2011). For this reason, open ponds and closed bioreactors have been applied long ago with high removal efficiencies (54–99%) (Gonçalves et al. 2017). In these systems, microbial consortium is typically developed, due to synergistic interactions of indigenous microorganisms, which may also affect nutrient removal and biomass production. Wastewater is an ideal feedstock for the cultivation of many heterotrophic bacteria which can establish complex interactions with microalgae and thereby positively or negatively influence algal growth, biomass composition and nutrient removal. Understanding these interactions is essential to explore algae-based wastewater treatment processes. Nevertheless, little research has been conducted on naturally selected symbiotic systems.

Furthermore, microalgae-bacteria processes could sustain a greenhouse reduction strategy by CO_2_ sequestration (Zhou et al. 2017) and reduce the aeration demand associated with conventional biological treatment. In addition, microalgae biomass has been considered an attractive source for production of bioenergy and biofuels for transport with many advantages over terrestrial crops and lignocellulosic feedstocks (Agostini et al. 2017).

Regardless of the specific advantages of open ponds and closed bioreactors, the main constrictions of the microalgae-based technologies are the complexity and associated cost of harvesting the small algal cells (< 30 µm), which may significantly contribute to the biological oxygen demand of the effluent (60–90%) (He and Xue 2010). The problem of algae harvesting from water can be solved by using attached algae; in fact, these attached algae are often observed as algal biofilm in the secondary effluent when sunlight hits on it (Lee et al. 2014). The attachment of microalgae could be passive to a bedding material, which is either completely or partially submerged to support biofilm development, or through entrapment (active) in gel matrices promoted by flocculent or chemical agents (Mohsenpour et al. 2021). Several studies have demonstrated and developed algal biofilm reactors for the efficient consumption of N and P from the wastewater and converting them into biomass (Yu et al. 2020) although these studies involved synthetic or diluted wastewaters and microalgae culture inoculation (Yang et al. 2018).

However, the application of membrane photobioreactor technology for wastewater treatment faces several drawbacks. Membrane fouling is one of the major constraints for its widespread application at full scale (Sheng et al. 2017). Small size of microalgae cells (3–30 µm) and release of biopolymers (algal organic matter and soluble microbial products) have been related to severe membrane fouling, which decreases process productivity (Liao et al. 2018). By analysing alternative cultivation strategies, bioflocculation can be enhanced to reduce membrane fouling.

In this study, the performance of organic and nutrient removal, biofilm growth and membrane fouling characterization were investigated in continuous operation of an attached spontaneous microalgae-bacteria consortium developed at lab scale, under controlled artificial lighting.

## Materials and methods

### Feedwater

Feedwater to lab bioreactor consisted of secondary effluent from a conventional activated sludge wastewater treatment plant (ASWWTP) without nutrient removal steps. Thirty litres of effluent was weekly collected from the ASWWTP and stored into a completely darkness feed tank in order to continuously supply at the unit. Its main characteristics are shown in Table 1.

**Table 1:** Main feedwater characteristics

Parameters	Units	Mean	Range
COD	mg·L^−1^	102	45–188
DOC	mg·L^−1^	22	13–26
pH	-	8.1	7.9–8.3
N-NH_3_	mg·L^−1^	51	24–66
N-NO_3_^−^	mg·L^−1^	0.2	0.1–0.6
P-PO_4_^3−^	mg·L^−1^	5.6	1.8–9.1
TSS	mg·L^−1^	22	3–115

### Membrane photobioreactor unit

**Fig. 1:**
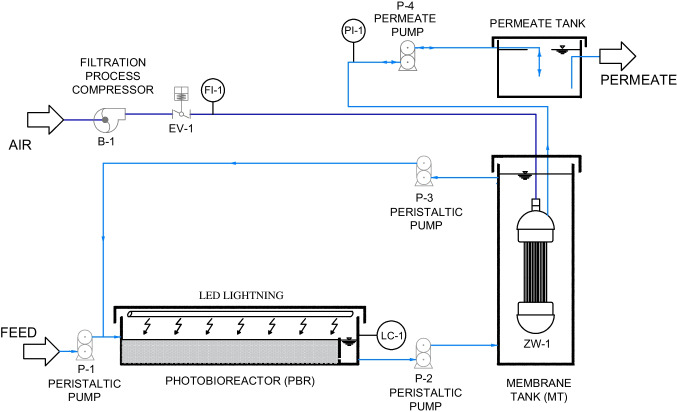
Experimental setup of the MPBR system

The membrane photobioreactor consisted of a combination of a photobioreactor (PBR) and a membrane tank (MT) equipped with an immersed hollow fibre membrane module (Fig. 1). The PBR was a rectangular plastic vessel of 13 L of total volume (effective volume of 1.8 L) and a transversal section of 0.09 m^2^. This reactor was filled with a bed of well-distributed glass Raschig rings of inert borosilicate glass. The physical characteristics of the carriers are described in Table 2. These immersed rings acted as media support for promoting preferential attached microalgae-based biomass growth instead of the walls or the suspended liquor. As the PBR was not inoculated, the biomass was developed from indigenous microorganisms present in the secondary effluent at room temperature (20 ± 2 °C). The hydraulic retention time (HRT) of PBR was 26 h. The PBR was closed in order to avoid sunlight incidence, and artificial light supplied by LED lightning was disposed in the inside cover. Light intensity was fixed at 490 µmol m^−2^ s^−1^ using a 12/12-h (light/dark) photoperiod cycle.

**Table 2:** Average carrier media and packed-bed characteristics

Parameters	Units	Value
Carrier media		
Material		Glass
Shape		Cylinder
External diameter	mm	9.60
Internal diameter	mm	7.20
Length	mm	11.70
Area	cm^2^	6.46
Sphericity	-	0.32
Packed bed		
Height	cm	2
Porosity	-	0.66
Number of carriers	-	2683

Feedwater was supplied to PBR by a peristaltic pump (P-1) (Easy-Load Masterflex, Cole-Parmer, USA), controlled by level (LC-1) (Aeman HSE-20987). The PBR supernatant was fed by a pulsing pump P-2 (Gamma L, ProMinent, Switzerland) at a flowrate of 0.069 L·h^−1^ to a vertical cylindrical membrane tank of a total volume of 2.2 L (MT). Recirculated supernatant from membrane tank was also fed by a secondary peristaltic pump (P-3) identical to P-1. Both feed streams were uniformly distributed to the bed by two perforated pipes of 8 mm diameter. The recirculation ratio of supernatant back from the membrane tank to the packed-bed biofilm was 1. An immersed hollow fibre membrane ZeeWeed®-1 (ZW-1) module (Suez Water Technologies & Solutions, Ontario, Canada) with a filtration surface of 0.047 m^2^ was vertically disposed into MT. Hollow fibre membranes show an external active layer of PVDF and average pore size diameter of 0.04 µm. The maximum transmembrane pressure recommended by the manufacturer is 62 kPa for filtration and 55 kPa for backwashing mode. Filtration process was carried out by vacuum created by a reversible pump P-4 (Micropump L-24295, 14–30 VDC, AxFlow, Stockholm, Sweden) which also allowed backwashing. Throughout the experimental time, ultrafiltration was carried out at constant permeate flux (*J* = 3 L·h^−1^·m^−2^). Furthermore, backwashing was applied every 7 min at constant conditions (*J*_B_ = 20 L·h^−1^·m^−2^ and *t*_B_ = 30 s) for fouling removal. Transmembrane pressure was measured by a transducer (PT-1) device and permeate was collected in a permeate tank, which allowed backwashing cycles and permeate discharge by direct overflow. The unit operated with aeration at 5 NL·min^−1^ during the backwashing. Control system of filtration cycle parameters (fluxes, pressures) was carried out by software DAQFactory (AzeoTech Inc., Ashland, OR, USA) run in a personal computer.

### Experimental methodology

Long-term experimental study was carried out feeding both secondary effluent from conventional ASWWTP and recirculated effluent from membrane tank. Hydraulic retention time of 1.1 day^−1^ was implemented, so this involved filtration cycles of 7 min at permeate flux of 3 L·h^−1^·m^−2^ and a backwashing time of 30 s at 20 L·h^−1^·m^2^, in order to ensure efficient nutrient removal (Honda et al. 2017). The bioreactor was run without biomass purge except for sampling. The whole study has been divided in two phases: initial biomass growth (phase I, first 1000 h of operation) and stationary period (phase II, from 1000 to 1500 h). Between both phases, the membrane was chemically cleaned for permeability recovery.

In addition, flux-step tests were applied to determine supernatant filterability characteristics and its variation along the study. A clean membrane module was used for each test. Then, step flux method was carried out at 4 different times of the operation: at initial time and after 384, 720, 1000 and 1500 h of operation. These experiments were run applying step of 5 L·h^−1^·m^−2^ every 15 min with intermediate backwashing for 30 s at 20 L·h^−1^·m^−2^. The limit conditions during the essays were 80 L·h^−1^·m^−2^ or TMP of 55 kPa to avoid critical membrane damage.

### Wastewater and suspension characterization

Along the study, feed and both effluents from PBR and MT were monitored 3 times a week. The parameters considered were chemical oxygen demand (COD), dissolved organic carbon (DOC), nitrogen compounds, turbidity, total suspended solids (TSS) and phosphate. COD, DOC, TSS, turbidity and pH were analysed according to the standard methods (APHA 2005). Turbidity was measured with a HACH 2100N turbidimeter (Hach, USA) and pH was determined employing a pH-metre WTW inoLab Level 1 (Xylem Inc., USA). DOC was measured with a SHIMADZU, TOCVCSH/TOC-VCSN device (Japan). The difference in DOC concentration between the filtrate of the suspension obtained through a 0.45-µm nitrocellulose membrane filter (HA, Millipore, Germany) and the ZeeWeed permeate was appointed as biopolymer clusters (BPC). Ammonium, nitrate, nitrite and phosphate were measured according to Spanish normalized guideline UNE-EN ISO 10304–2 employing a METHROM 882 Compact IC plus chromatograph. To quantify the amount of non-flocculating microorganisms, the samples of biomass were centrifuged at 1300 g for 2 min and the supernatant turbidity was measured (Ng and Hermanowicz 2005).

Regarding PBR biomass, it consisted of a biodiverse microbial consortium whose properties could be determined on its filterability and, as consequence, on membrane fouling characteristics and mechanisms, but also on its possible further valorization. For elemental analysis, a FlashEA 1112 Elemental Analyzer (Thermo Fisher Scientific, USA) was used.

### Isolation and characterization of species

To isolate and characterize microbial species, liquor samples of 10 mL, rings and biofilm accumulation on the membrane of the MPBR were collected at different stages of the process. All samples were stored at 22 °C in a culture room. Samples were inoculated using the streaking strain method in PDA (Scharlab) and MM2 (starch, 10 g·L^−1^; yeast extract, 4 g·L^−1^; peptone, 2 g·L^−1^; bacteriological agar, 10 g·L^−1^; supplemented with 5% filtered seawater) agar plates. Inoculated agar plates were incubated for at least 2 days at two different conditions: (a) 24 °C under dark; and (b) 22 °C under a 16/8-h (light/dark) photoperiod cycle. Once pure cultures were obtained, laboratory-scaled cultures were conducted. Ten millilitres of cultures contained in 30-mL flasks with PDB and MM2 liquid media was inoculated with the different strains of bacterial and yeast isolates. Fungal isolates were kept in agar plates. All isolated strains are kept at − 80 °C.

### DNA extraction, PCR amplification and sequencing

These steps were carried out at the Genomic Service (General Research Support Services of La Laguna University). Yeast and fungal genomic DNA were purified using the E.Z.N.A Plant DNA kit (Omega Bio-tek), according to the manufacturer’s instructions, but with the addition of 10 mg of Proteinase-K to lysis buffer and increasing the incubation time 30 min at 65 °C. Bacterial genomic DNA was extracted from liquid cultures after centrifugation (5 min at 18,000 × g) and resuspension of cell pellets in 200 µL of lysis buffer (40 mM Tris–HCl pH 7.8, 20 mM sodium acetate, 1 mM EDTA, 1% SDS, 0.04 µg·µL^−1^ RNase-A (Promega), 0.04 µg·µL^−1^ lysozyme (Thermo Fisher Scientific)). Samples were incubated for 5 min at RT and sequentially treated with 5 M NaCl and chloroform. After centrifugation (18,000 × g, 2 min), the aqueous phase was recovered, and DNA was precipitated with cold absolute ethanol and washed twice with 70% ethanol. DNA pellets were dried at RT and then suspended in 50 µL of H_2_O.

Bacterial 16S ribosomal region was amplified using universal primers 27F (5′-TAGAGTTTGATCMTGGCTCAG-3′) and 1492R (5′-TACGGYTACCTTGTTACGACTT-3′), described by Weisburg et al. (1990). In the case of fungi and yeasts, amplification and sequencing for species identification were conducted on ITS1-2 region using generic primers ITS1 (5′-TCCGTAGGTGAACCTGCGG-3′) and ITS4 (5′-TCCTCCGCTTATTGATATGC-3′) (White et al. 1990). Commercial PCR kit AmpONE Taq DNA polymerase (GeneAll Biotech) was used for amplification, and the PCR-amplified products were checked for the presence of a single band of the expected size by standard agarose electrophoresis. PCR products were purified with the EXO-SAP-IT kit (Affymetrix-USB) following the manufacturer’s instructions. Sanger sequencing was carried out using the BigDye™ Terminator v3.1 Cycle Sequencing Kit (Thermo Fisher Scientific) and 3500 Series Genetic Analyzer automatic sequencer (Thermo Fisher Scientific). The SeqScape (Thermo Fisher Scientific) software was used for the analysis of sequencing data and filtering out low-quality reads.

### Phylogenetic analysis

DNA sequences were assembled, and multiple alignments were performed using CLUSTAL W implemented in MEGA7 (Kumar et al. 2016) software package (v.7.0.21) in order to obtain the identity of the isolated strains by comparison with other DNA sequences in BLAST (Camacho et al. 2009).

## Results and discussion

### Initial growth of microalgal-bacterial biofilm

**Fig. 2:**
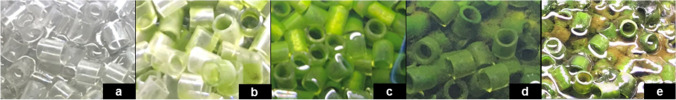
Biofilm development with microalgal-bacterial consortia on carrier media. **a** 0 h, **b** 380 h, **c** 550 h, **d** 720 h, **e** 1200 h

**Fig. 3:**
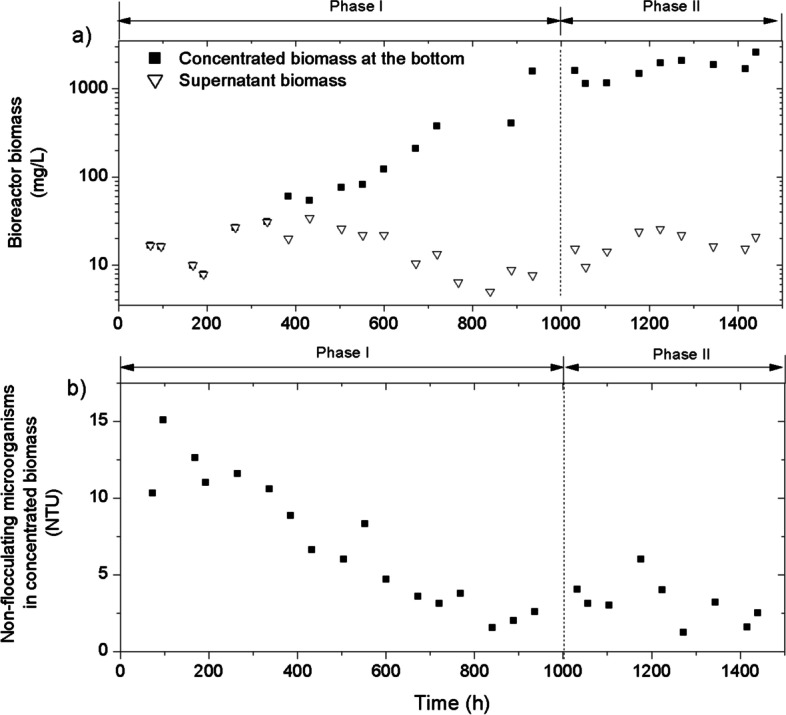
Evolution of **a** concentrated and supernatant biomass in the bioreactor and **b** non-flocculating microorganisms in concentrated biomass

Since the photobioreactor started without inoculum, biofilm was developed from the native microorganisms entered with the feedwater. A significant variability on feedwater characteristics was reported along the study, especially for TSS, turbidity and organic matter contents (Table 1). It could be due to the fed wastewater which was the secondary effluent from a conventional WWTP affected by unavoidable fluctuations of sewage characteristics and WWTP performance itself. After 380 h of operation, biofilm started to be developed in the submerged carrier media (Fig. 2b). The biofilm gradually colonized the carriers reaching the average values of 4.3, 6.3 and 7.3 g·m^−2^ at operating times of 550, 720 and 1200 h, respectively (Fig. 2c, d and e). At the same time, the biofilm was progressively detached and accumulated at the bottom of the bioreactor. Figure 3a compares the evolution of concentrated (at the bottom of the bioreactor) and supernatant biomass concentrations in the bioreactor. An appreciable increment of concentrated biomass can be observed after 380 h of operation (Fig. 3a), consistent with the appreciable growth of biofilm at the same period. The amount of suspended biomass throughout the experimental period is very limited, as can be seen by the low concentration in the supernatant of the bioreactor (5–34 mg·L^−1^). Therefore, the advantage of the glass carrier media was demonstrated, since it allowed an adequate growth of the biofilm without excessive adherence, so that it progressively detached and accumulated at the bottom of the bioreactor. Thus, the packed-bed biofilm photobioreactor allows working at high concentrations, achieving good contact without agitation between the liquid medium and the biomass, as occurs with conventional photobioreactor systems. As a consequence of the above, the use of this type of photobioreactors would allow for a saving in energy consumption.

The accumulated biomass increased exponentially until it reached values close to 1500 mg·L^−1^ at about 1000 h of operation. Thereafter, the rate of biomass accumulation slowed down significantly to 54 ± 18 mg·L^−1^·day^−1^. This decrease in productivity can be attributed to light attenuation at high biofilm density (Kesaano and Sims 2014). Nevertheless, the obtained value is comparable with those typically reported for membrane photobioreactors (Luo et al. 2017). Therefore, the operation time of 1000 h was selected as the necessary period for the start-up of the system and was identified as experimental phase I.

**Fig. 4:**
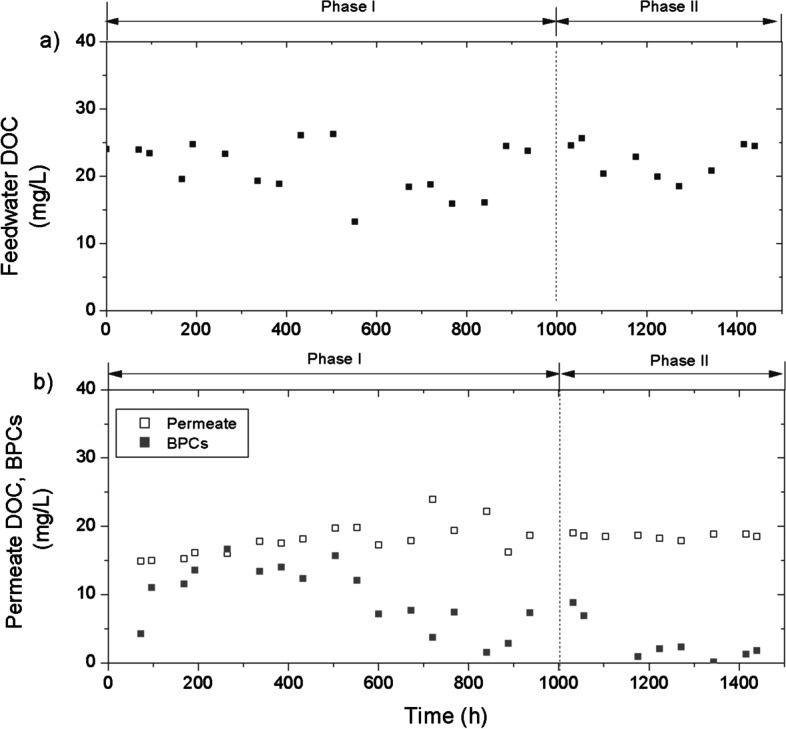
Evolution of **a** feedwater DOC (mg·L^−1^) and **b** supernatant BPCs and permeate DOC (mg·L^−1^) during the experimental time

The progressive acclimatization of the biomass during phase I can also be observed in the evolution of the characteristics of the biomass accumulated at the bottom of the bioreactor. If the degree of flocculation (amount of non-flocculates in the supernatant after centrifugation) is analysed, it is observed that it increased with the operation time until its stabilization at the end of phase I (Fig. 3b). According to indigenous biomass growth, a microalgae-bacteria consortium was expected to be developed into the bioreactor. It has been reported that the synergistic support of bacteria and microalgae enhanced bioflocculation (Fallahi et al. 2021), consistent with the observed results in our study. The development of heterotrophic and nitrifying bacteria in the biofilm can be also assessed by analysing organic matter and nitrogenous compound oxidization in the bioreactor, respectively. It should be noted that the dissolved oxygen was always maintained at high levels (> 8 mg·L^−1^) due to the microalgae activity. Figure 4 shows the evolution of dissolved organic matter (DOC) in the feedwater, the supernatant (i.e. biopolymer clusters, BPCs) and the permeate during experimental run. Feedwater DOC ranged between 13.2 and 26.2 mg·L^−1^, with an average of 21.6 ± 3.5 mg·L^−1^. BPCs are organic colloidal substances, formed from soluble microbial products generated during biomass growth and decay, which have been related to bioflocculation process (Fallahi et al. 2021). An accumulation of these substances can be observed during the first 500 h of phase I, but then it progressively decreased (Fig. 4b). In fact, very low values of BPCs (1.8 ± 0.8 mg·L^−1^) were found at the end of phase II. Therefore, the observed trend can be related by a progressive biodegradation/adsorption of BPCs as the biofilm developed. In turn, this tendency can be associated to heterotrophic growth, which can degrade soluble microbial products (Barker and Stuckey 1999). The biodegradation/adsorption of BPCs is enhanced by working at high SRTs which allow for higher concentrations, and the use of carriers promotes biofilm growth. The type of photobioreactor proposed in this work allows a lower concentration of BPCs with respect to other systems reclaiming similar feedwater but with a typical bubbling column configuration (Segredo-Morales et al. 2022).

**Fig. 5:**
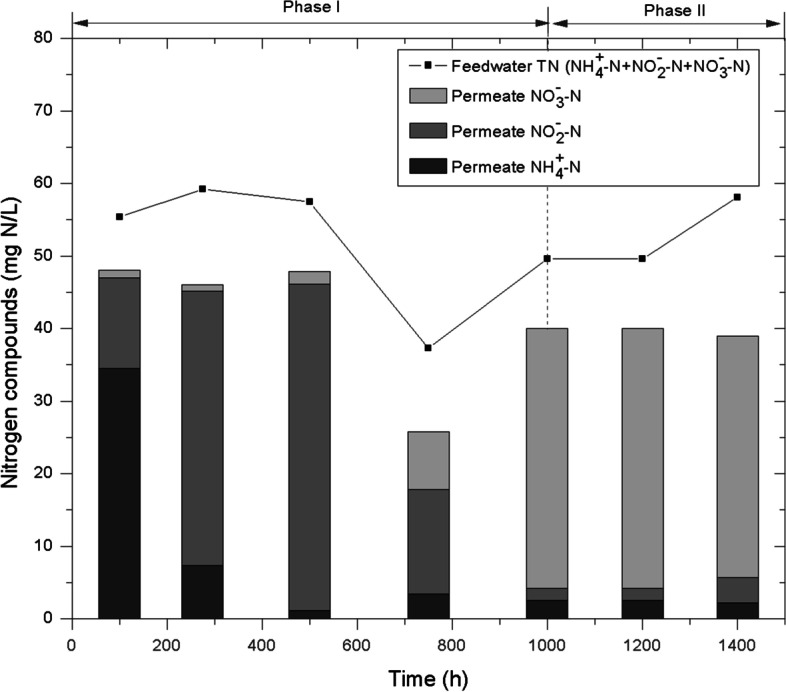
Nitrification process evolution in the bioreactor

Consistently, stable values of permeate DOC were observed in phase II (Fig. 4b). Regarding nitrification, it involves the development of nitrifying bacteria for converting ammonium to nitrite and then nitrite to nitrate. The first step of conversion required around 500 h and the second one at approximately 1000 h (Fig. 5). Both processes are related with nitrifying bacteria development, a type of chemolithotrophic organism whose growth can be seriously compromised by expelled metabolites by microalgae (Choi et al. 2010). This inhibition is consistent with the large period required to achieve a complete nitrification in the present study compared to that previously reported of 480 h in a tertiary membrane bioreactor fed with similar wastewater (Díaz et al. 2016). Nevertheless, steady nitrification was achieved once the biofilm stabilized in phase II.

### Process performance and biomass characteristics during the stabilized period

As previously stated, effluent organic matter was practically stable, around 43–61 mg·L^−1^, expressed as chemical oxygen demand (COD), which involved removal efficiencies of 56 ± 5% (Table 3). The reported effluent COD values were comparable with those achieved by Vera et al. (2014a, b) during performance of a tertiary membrane bioreactor fed with similar wastewater. Regarding nitrogen compound removal, it can be generally associated to three different phenomena: nitrification, denitrification and bio-assimilation. In the case of microalgae, these microorganisms can absorb ammonia directly into the cell, then accumulate it in the form of amino acids and finally use them for its metabolism as reported (Kim et al. 2013). In the present study, average nitrogen removal was 12 ± 2% (Table 3). This total nitrogen removal percentage is lower than those reported in previous studies (Ruiz-Martinez et al. 2014; Luo et al. 2017), but in those studies, synthetic wastewaters were fed, while in the present work, diversity of natural biota and their competition for resource uptake are completely different and uncertain. However, the system achieved a complete nitrification of the effluent without an external air supply only through the action of biofilm-associated microbial community (Fig. 5). Low PO_4_^3−^-P removal was also obtained, with an average of 20 ± 6%. Calculated removal rates were 4.2 mg·L^−1^·day^−1^ and 0.8 mg·L^−1^·day^−1^ for nitrogen and phosphorus, respectively, which resulted in ratios of eliminated nutrient to increased biomass of 7.8% and 1.5%, respectively. Nitrogen ratio was closely determined by elemental analysis (6.4 ± 1.3%, Table 3). Similarly, phosphorus ratios were within the range reported in the literature (Ruiz-Martinez et al. 2014). Therefore, results revealed that biomass assimilation was the main removal mechanism for the nutrients.

**Table 3:** Photobioreactor treatment performance and elemental analysis of the biofilm during experimental phase II

Parameter	Units	Value
Photobioreactor treatment performance		
COD removal	%	56 ± 5
P-PO_4_^3−^ removal	%	20 ± 6
Nitrogen removal (N-NH_4_^+^ + N-NO_3_^−^ + N-NO_2_^−^)	%	12 ± 2
Nitrogen removal rate	mg·L^−1^·day^−1^	4.2 ± 1.1
Phosphorus removal rate	mg·L^−1^·day^−1^	0.8 ± 0.4
Biomass productivity rate	mg·L^−1^·day^−1^	54 ± 18
Elementary analysis (wt, dry and ash-free basis)		
C	%	45.8 ± 2.1
H	%	6.7 ± 0.4
N	%	6.4 ± 1.3

The data are expressed as mean ± standard deviation

### Microbial community

The analysis of the composition of microbial communities is key to understand and optimize wastewater treatment processes (Dueholm et al. 2022). Under the experimental conditions tested, biofilm-associated microbial community consisted of a complex consortium of Gram-positive and Gram-negative bacteria and fungi, together with the photosynthetic microalga *Tetradesmus obliquus* (Tables 4 and 5), which can contribute to carbon consumption and nitrogen removal. *T. obliquus*, also known as *Scenedesmus obliquus*, is a versatile species with fast growth rate and highly resistant to extreme conditions (Di Caprio et al. 2018). Its ability to grow in nitrogen- and phosphorus-rich wastewater as well as its high resistance to temperature and irradiance confirms this microalga as suitable for emerging environmental applications (Renuka et al. 2016; Oliveira et al. 2021). Those include wastewater treatments in which *T. obliquus* had showed better performance than *Chlorella vulgaris* and *Oocystis minuta* (Ajala and Alexander 2020). Bacteria play a key role in the biological processes of nitrification and denitrification of treated waters. That is the case with *Comamonas* spp. *C. nitrativorans* was identified as a new species isolated from a denitrifying reactor to efficiently reduce nitrate to N_2_ (Etchebehere et al. 2001), as well as *C. denitrificans* sp. nov., isolated from activated sludge (Gumaelius et al. 2001). *Bacillus* spp. are Gram-positive bacteria. They are ubiquitous in nature and isolated from different niches in the environment. *Bacillus* spp. can modulate physical and chemical water parameters which include pH, conductivity, chemical oxygen demand, dissolved oxygen, biological oxygen demand, alkalinity, phosphates, or nitrogenous species among others, as well as reduction in pathogenic microbes (Kuebutornye et al. 2019). *Pseudomonas* species, in particular *Pseudomonas putida* Y-9, has been described for its excellent capabilities to efficiently remove ammonium, nitrate and nitrite through heterotrophic nitrification and aerobic denitrification, even at low temperature (Banerjee et al. 2017). Both *Bacillus* and *Pseudomonas* species have been described for their properties as plant growth-promoting bacteria and as suppressors of plant pathogens (Radhakrishnan et al. 2017; Maes et al. 2020). These mechanisms open new alternatives for bio-control strategies. Bacteria also contribute to biodegradation of organic compounds in wastewater treatments. For instance, species of genus *Sphingopyxis* have been considered of interest not only for their ability to survive under extreme environments but also for their potential to degrade xenobiotics and other environmental contaminants which may pose serious threat to human health (Sharma et al. 2021). That is also the case with *Microbacterium chocolatum*, a symbiotic bacterium associated with *Chlorella* strains to contribute to nutrient removal and remediation of wastewater (Wu et al. 2019; Nagarajan et al. 2020). The presence of pathogenic bacterial species such as *Escherichia coli*, *Atlantibacter hermannii*, *Escherichia hermannii* and *Mycobacterium* sp. can be attributed to the particular characteristics of feedwater.

**Table 4:** List of bacterial and microalgal isolates

Strain ID	Isolation medium	Most closely related type strain^a^
Bacteria		
B1_B03	PDA	*Bacillus* sp.
B1_B16	PDA	*Bacillus* sp.
B1_B19	PDA	*Bacillus* sp.
B1_B20	PDA	*Mycobacterium* sp.
B2_B01	MM2	*Escherichia coli*
B2_B02	MM2	*Pseudomonas* sp.
B2_B05	MM2	*Bacillus* sp.
B2_B06B	MM2	*Sphingopyxis chilensis*
B2_B07	MM2	*Comamonas* sp.
B2_B08	PDA	*Pseudomonas putida*
B2_B10	PDA	*Mycobacterium* sp.
B3R_B18	MM2	*Microbacterium chocolatum*
B3W_B09	MM2	*Atlantibacter hermannii*
Microalgae		
B3R_B17	MM2	*Tetradesmus obliquus*

^a^The corresponding type of strain had a level of similarity greater than or equal to 99%

**Table 5:** List of yeast and fungal isolates

Strain ID	Isolation medium	Most closely related type strain^a^
Yeast		
B1_B04	PDA	*Rhodotorula mucilaginosa*
Fungi		
B1_B13	PDA	*Acremonium* sp.* (*Cephalosporium*)
B1_H03	PDA	*Penicillium janthinellum*
B1_H04	PDA	*Penicillium bilaiae*
B1_H05	PDA	*Cladosporium ramotenellum*
B1_H06	PDA	*Fusarium solani*
B1_H07	PDA	*Malassezia globosa*
B2_B15	PDA	*Exophiala mesophila*
B2_H01	MM2	*Purpureocillium lilacinum*
B2_H02	MM2	*Mucor circinelloides*
B2_H10	PDA	*Penicillium janthinellum*
B2_H13	PDA	*Purpureocillium lilacinum*
B3R_H11	MM2	*Trichoderma asperellum*

^a^The corresponding type of strain had a level of similarity greater than or equal to 99%

Similarly, the use of filamentous fungi has been recognized of interest for different wastewater sludge treatments such as bioflocculation and pathogen reduction, as well as degradation and removal of toxic compounds (More et al. 2010). These properties have been studied for certain species of genus *Penicillium*, *Aspergillus* and *Trichoderma* (More et al. 2010). Among the identified species, one study on performance of *Rhodotorula mucilaginosa* reveals the ability of this yeast to degrade some phenolic compounds and to grow on olive mill wastewater (Jarboui et al. 2012). However, except for the environmental pathogen strain *Fusarium solani*, most of the isolated fungal strains are human pathogens which spores can be easily transferred through feedwater into the system.

### Fouling analysis

#### Membrane process performance in long-term trials

**Fig. 6:**
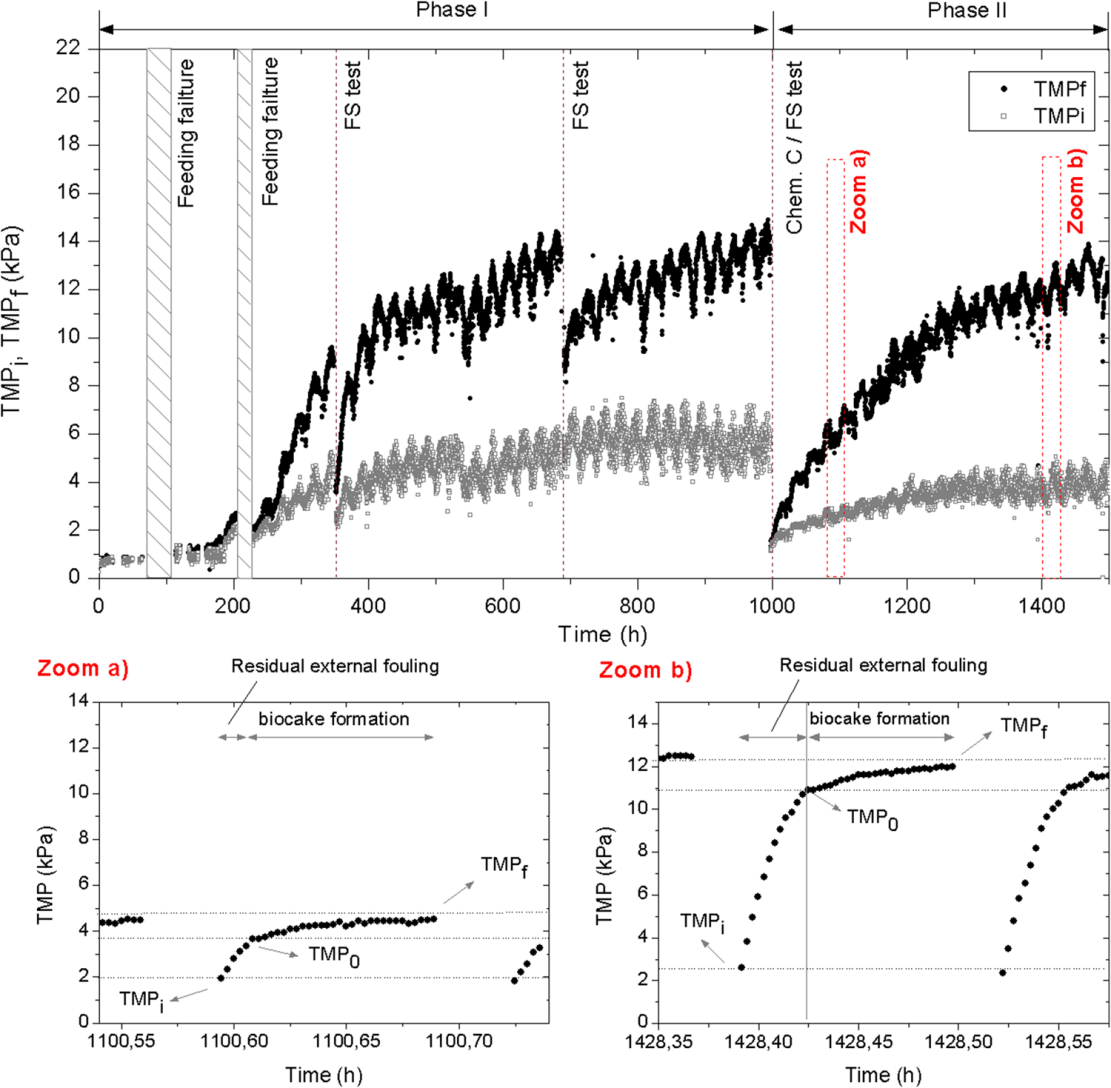
Evolution of initial (*TMP*_*i*_) and final (*TMP*_*f*_) transmembrane pressures during the experimental period; *TMP* profiles during consecutives filtration cycles at zoom (a) 1100 h and zoom (b) 1428 h. FS test, flux-step tests; Chem. C., chemical cleaning

Conventional filtration operation of the MPBRs typically involves periodical backwashing cycles for mitigating membrane fouling (Luo et al. 2017). Figure 6 shows initial and final transmembrane pressures (*TMP*_*i*_ and *TMP*_*f*_, respectively) during both experimental phases. While the former was associated with internal residual fouling that cannot be removed with physical cleanings, the latter was due to external fouling. Several feeding failure events occurred during the first phase without significantly affecting membrane performance. Then, the operation was stopped two times for conducting flux-step tests (FS test). In both cases, a significant decrease *TMP*_*f*_ was observed, which related to the reversible nature of the external fouling. As expected, ceasing the filtration for a short period of time (i.e. applying a relaxation) significantly reduced external fouling, as widely reported in membrane bioreactors (Wang et al. 2014). Consistently, the effect on residual internal fouling (i.e. *TMP*_*i*_) was less noticeable. At the end of phase I (1000 h), another flux-step test was conducted. In addition, the membrane module was chemically cleaned, recovering its initial values.

Membrane fouling development was assessed by following *TMP* trends. *TMP*_*f*_ profile suggested a dynamic behaviour where, after a lag period of about 200 h, transmembrane pressure sharply increased and then followed a continuous growth (Fig. 6). This behaviour can be due to a biofilm formation, which has been reported as a major concern in MPBRs (Liao et al. 2018). Consistently, *TMP*_*f*_ started to grow from the beginning of phase II, without a lag period, confirming the relationship between the microbiological activity in the photobioreactor and the fouling observed in the membrane. A deeper analysis of the *TMP* trends during the filtration cycles (Fig. 6, zoom (a) and zoom (b)) showed that *TMP*_*f*_ was developed through two consecutive stages: an initial sharp increase followed by a linear rise. Therefore, a value of *TMP* (*TMP*_*0*_) can be distinguished, which separates both fouling stages. The initial stage can be related to the inefficiency of backwashing, where the biofouling layer is fragmented into particle clusters which are only partially re-dispersed from the membrane pore structure, as has been recently proposed (Lohaus et al. 2020). Therefore, the remaining clusters are expected to enhance clogging when filtration resumes. Results also showed the predominant role of the residual external fouling in the present study, which significantly increased with operating time (Fig. 6 zoom (a) and zoom (b)). Similar results have been reported in dead-end ultrafiltration of model foulants (Ye et al. 2011). Regarding the second fouling stage (i.e. the linear *TMP* rise), it can be related to subsequent newly foulant deposition forming a biocake layer, which was mainly influenced by permeate flux and supernatant BPC content (this will be analysed later in the ‘Effect of supernatant characteristics on fouling propensity’ section). Due to low values for both parameters in the tested conditions, consistent relatively low fouling rates were observed. Nevertheless, it appears that the inefficiency of backwashing for re-dispersing the deposited foulants increased the external fouling along with consecutive filtration cycles.

**Fig. 7:**
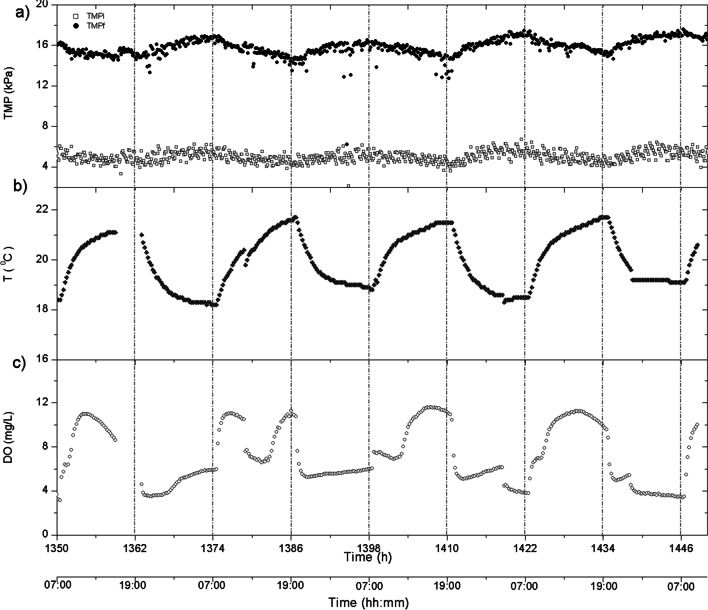
Evolution of **a** initial (*TMP*_*i*_) and final (*TMP*_*f*_) transmembrane pressures, **b** temperature and **c** dissolved oxygen (DO) during several consecutive days in phase II

*TMP*_*i*_ profile presented several similarities with the trend observed with *TMP*_*f*_, such as lag period followed by a rapid increase in phase I (Fig. 6). Nevertheless, this type of fouling stabilized at around 800 h, revealing that the backwashing balanced the internal fouling growth. In this sense, lower stabilized *TMP*_*i*_ values were obtained in phase II, where lower BPCs were observed (Fig. 4b). This is consistent with previous studies that reported that biofilm growth is enhanced over a previously organic coated membrane surface (Liao et al. 2018). In addition, a substantial effect of photoperiod pattern on *TMP*_*i*_ profile was observed (Fig. 7a). A clear relationship with the temperature profile, regardless of the photosynthetic activity of oxygen production, was found (Fig. 7b and c). This was probably due to the impact of temperature on permeate viscosity, which increases membrane permeability.

#### Effect of supernatant characteristics on fouling propensity

The influence of the supernatant characteristics on membrane fouling was studied by flux-step tests, which a clean membrane was subjected to increasing flux steps higher than the one used in the long-term tests. Tests were performed at different operating times (0, 350, 687, 1000 and 1500 h) by filtering the supernatant from the bioreactor. Due to the short duration of the tests, only the influence on the formation of the biocake can be analysed.

**Fig. 8:**
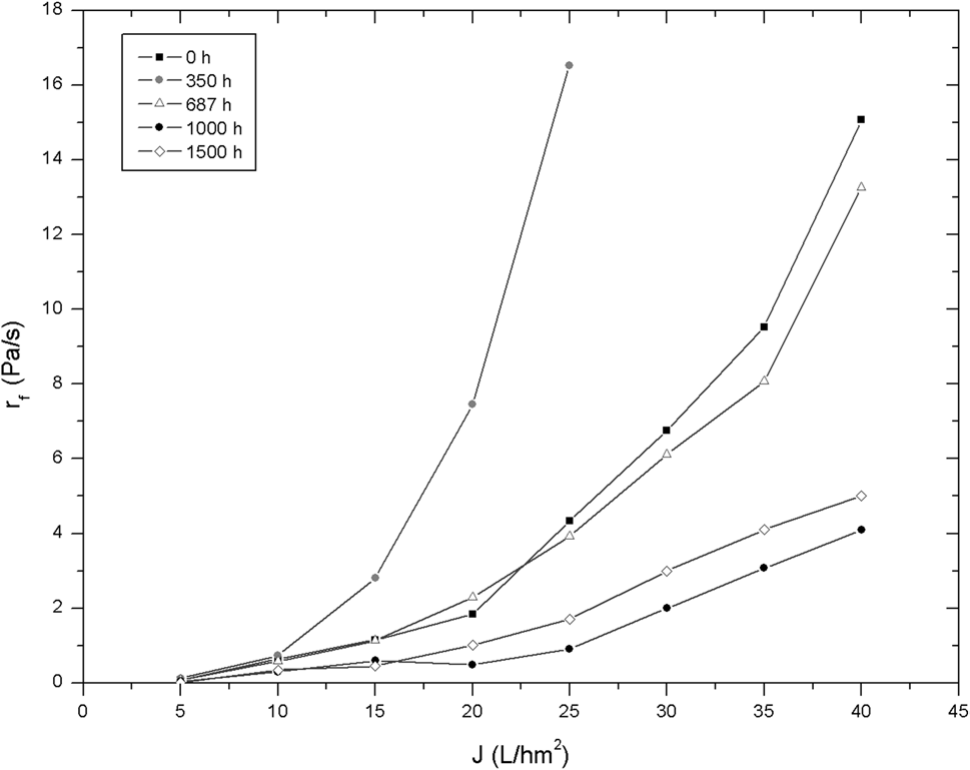
Membrane fouling rates against permeate flux during the flux-step trials, obtained by filtering the supernatant of the bioreactor taken at different experimental times

The slope of the *TMP* against time (*r*_*f*_ = d*TMP*/d*t*) at each step represents the fouling rate due to fouling deposition, similar to that reported in many other studies (Boonchai and Seo 2015). Figure 8 shows the fouling rates at different permeate fluxes. At low operating fluxes (*J* < 10 L·h^−1^·m^−2^), membrane fouling was not affected by the characteristics of the suspension, presenting low and similar fouling rates. On increasing* J*, *r*_*f*_ sharply increased. This behaviour is explained by the sharp increase of particle deposition rates at high fluxes. In addition, supernatant characteristics showed a noticeable effect on fouling rates when the flux was increased. The lowest *r*_*f*_ sharply increase, at moderate and high filtration fluxes, was reported when the BPCs were stabilized (> 1000 h of operation time). Moreover, the maximum fouling rates reported for each filtration flux imposed were reached at 350 h of operation time, which coincided with the maximum BPC concentration in the supernatant. The fouling rates show similar behaviour at 0 h and 687 h of operation time, where BPC content was moderate (4–8 mg·L^−1^). Previous studies have also identified BPCs as the main fouling indicator (Díaz et al. 2016). Deposition of biopolymers occurs during filtration on membrane surface and pore surface to form a viscous and elastic gel layer (Liao et al. 2018). Many studies have shown the fouling rate in MPBR decreases when the organic matter concentration is reduced (Liao et al. 2018). The results are consistent with the long-term trials revealing the crucial role of BPCs in membrane fouling due to the subcritical conditions imposed in the filtration process where the predominant mechanism is the growth of a biofilm.

## Conclusions

Combined packed-bed photobioreactor with ultrafiltration membrane system was assessed for advanced wastewater treatment. A microbial biofilm was successfully developed from native microorganisms in glass Raschig rings used as supporting material, which significantly limited the suspended biomass in photobioreactor. After a start-up period of 1000 h, the system achieved a stable biomass productivity (54 mg·L^−1^·day^−1^) and minimized the supernatant biopolymer content (< 1.8 mg·L^−1^). In these stable conditions, green microalgae *Tetradesmus obliquus* and several groups of heterotrophic nitrification–aerobic denitrification bacteria and fungi were identified. Moderate organic matter (56 ± 5%) and nutrient removal efficiencies were also achieved (12 ± 2 and 20 ± 6% for nitrogen and phosphorus, respectively). Despite the low suspended biomass and biopolymer content in the bioreactor supernatant, a significant membrane fouling was achieved, mainly due to biofilm formation, which was not effectively mitigated by air-scouring aided backwashing.

## Data Availability

Data and results will be available on request.
